# School Bullying in Urban China: Prevalence and Correlation with School Climate

**DOI:** 10.3390/ijerph14101116

**Published:** 2017-09-25

**Authors:** Ziqiang Han, Guirong Zhang, Haibo Zhang

**Affiliations:** 1 Institute for Disaster Management and Reconstruction, Sichuan University, Chengdu 610000, China; hanziqiang@scu.edu.cn; 2 Center for Crisis Management Research, Tsinghua University, Beijing 100084, China; 3 School of Public Administration, Central South University, Changsha 410083, China; zhangguirong@csu.edu.cn; 4 School of Government, Nanjing University, Nanjing 210008, China

**Keywords:** school bullying, victim, perpetration, witness, China, school climate

## Abstract

School violence and bullying in China is under investigated, though it has become a national concern recently. Using updated national representative survey data collected in 2016 from seven provinces across China, covering students from all pre-college school types (primary, middle, high and vocational schools), this paper analyzes the prevalence of school bullying and the correlation with several school attributes. The incidences of reported bullying, bullying others and witnessing bullying are 26.10%, 9.03% and 28.90%, respectively. Primary school students are more likely to be involved in bullying behaviors. Students from elite schools (leading schools) are also more likely to be involved. Relation with teachers, relation with peers and perceived academic achievement are protective factors. Being a boy is the only significant predictor of school bullying among the family and demographic characteristics used. The results highlight the importance of school climate on preventing school violence and bullying, and a whole-school intervention approach is needed for future intervention.

## 1. Introduction

School bullying is a worldwide phenomenon and a growing but relatively new research area with problems, disagreements and unsolved issues since its emergence in the late 1960s [1]. School bullying can cause post-traumatic stress disorder (PTSD) [2], low self-esteem [3], psychosocial problems such as depression, loneliness, anxiety, emotional issues [4,5], somatic symptoms [6], poor academic achievement, psychosocial adjustments [7], deviant behaviors [8], long-term mental health problems and even violent [9] or suicidal behaviors [10,11]. Both victims and perpetrators of school bullying can experience these consequences, with the potential of reciprocal influences [12]. Prior cross-cultural studies on school bullying indicate that incidences of bullying in collectivism-oriented countries like China are much lower than individualism-oriented countries such as the United States [13], and school bullying is a topic with both universal patterns and culturally sensitive characteristics [14]. Thus, examining school bullying among Chinese adolescents could not only contribute to our understanding of school violence and bullying in China but also add valuable knowledge in this field worldwide.

Recent school violence cases in China, such as the attack at Beijing’s Zhongguancun No. 2 Primary School where several students bullied one boy by pouring a trashcan full of used toilet paper and feces on his head, renewed the severity of school violence in China [15]. These extreme cases were amplified by the internet and social media, and attracted lots of attention from the public and authorities [16]. The Chinese education and legal authorities have pledged to fight against school bullying and violence since then, and several anti-bullying and anti-school violence policies have been released with strong actions. In 2016, 1180 people involved in school bullying and violence were arrested, including both adults and juveniles [17]. However, in contrast to the public’s concern and huge policy needs, the knowledge of school bullying and violence in China is so under investigated, even the definition of school bullying and school violence are not clearly defined or differentiated in the related policies. Also, due to the absence of rigorous academic studies with national representativeness, the mass media’s distorted [1] report that 87% of the 1st to 9th grade students reported being bullied and 49% reported bullying others [18], was suspicious and misleading. Thus, there is a need for rigorous academic investigation to help the public and authorities to understand the real picture of school bullying in China, and further implement preventions and interventions.

Empirical studies on school bullying in China are very limited compared to the volume of research in Western contexts. In a review of school bullying in Greater China conducted by Chan and Wong in 2015, the self-reported prevalence of victimization ranged from 2% to 66% in Mainland China, 24% to 50% in Taiwan, 20% to 62% in Hong Kong and 26% to 78% in Macau. Meanwhile, the self-reported perpetration in Mainland China ranged from 2% to 34%, 40% to 68% in Taiwan, 19% to 56% in Hong Kong and 16% to 46% in Macau [19]. These differences of prevalence indicate the ambiguousness and uniqueness of the school bullying phenomena in Mainland China. Besides the studies from Hong Kong [20], Taiwan [21,22] and Macau [23,24,25,26], which are relatively different from Mainland China, most of the school bullying studies from Mainland China only used data from a small area, such as Beijing [27], Fujian [28], Guangdong [29,30,31], Xi’an [32], or unidentified central [33] or southwest China [34]. Two studies using national data were identified. One was a study only covering the middle school students from four cities, and the data was collected between 2002 and 2003 as a part of WHO’s Global School-based Health Survey project [7]. Another used more recent data collected between 2011 and 2012, but only students in high schools were surveyed [8]. Thus, the current empirical studies on school bullying in China mainly included part of the youth in terms of geographical distribution or types of schools (e.g., middle or high school). This paper contributes to current knowledge by analyzing updated representative national data collected at the end of 2016, covering a wide geographical area of China and all types of pre-college schools—primary school, middle school, high school and vocational school—to unveil the overall school bullying faces in Mainland China.

Besides the demographic and socioeconomic variables, family related attributes such as the change of family structure, parent-children relationship and inter-parental violence; school climate such as the relation with peers, school connectedness and school environment, and teacher involvement; and community and societal influences such as exposure to media violence, neighborhood environment, cultural norms and beliefs, and religious affiliation are the common risk factors in bullying perpetration and victimization [12]. Evidence from Australia [35], New Zealand [36], the United States [37,38,39] and China [40] indicate that a positive school climate plays an important role in reducing school bullying, or a moderate role in mitigating the negative impacts of bullying behaviors [41].

Since most of the previous studies on school bullying in China mainly examined middle school or high school students from one region, and primarily focused on the prevalence and consequences of being bullied, our analysis covers adolescents from all types of pre-college schools, with multi-perspectives from bullying victims, perpetrators and bystanders (witnesses). What’s more, we grouped the students into non-victim and non-perpetrator, victim-only, perpetrator-only and victim-perpetrator categories [24,42,43], and analyzed the correlations between several school attributes and the probability of being victims or perpetrators with the control of basic family and demographic variables. We hypothesize that:

1. Primary school students would have higher school bullying prevalence than middle school and high school students;
2. Vocational school students would have higher school bullying prevalence than students from other schools;
3. Students from elite schools (leading schools) would have lower prevalence of school bullying than the students in non-leading schools;
4. With better relation with teachers, a student would have a lower likelihood of being a bullying victim or perpetrator;
5. With better relation with peers, a student would have a lower likelihood of being a bullying victim or perpetrator;
6. With better academic performance (perceived GPA), a student would have a lower likelihood of being a bullying victim or perpetrator.

## 2. Methods

### 2.1. Participants and Sampling

The School Safety Research Committee of the China Emergency Management Association organized a national school bullying survey at the end of 2016. Seven provincial capital cities from Liaoning, Beijing, Jiangsu, Hunan, Guizhou, Guangdong and Gansu were purposively selected due to their geographical locations within China. Within each city, one primary school, one middle school, one high school and one vocational/technical school were conveniently selected, considering the availability of connections. The representation criteria of the schools were taken into consideration in the school selection process. Within each middle school, high school and vocational school, one class in each grade was randomly selected and all the students within that class were surveyed. Within the primary schools, only grade four to six students were selected to participate in the survey. All these questionnaires were finished by the students themselves, with technical help from our data collection team members who were trained graduate students, and the survey was anonymous. In total, 3777 questionnaires from 28 schools (4 schools/province*7 provinces) were collected and 3675 were included for analysis because the other 102 observations had missing values, and thus they were dropped.

### 2.2. Measurements

#### 2.2.1. Bullying

The school bullying questions were mainly adopted from the School Crime Supplement (SCS) to the National Crime Victimization Survey developed by the United States’ National Center for Education Statistics [44]. The definition and measurement of school bullying varied in different countries and cultures, and many research tools have been developed by researchers worldwide [45]. The Olweus school bullying measurement and intervention tool is one of the most widely adopted [1]. However, it is not free for academic use. Moreover, we found that the expressions of many questions used the term “bullying”, which is not appropriate in the Chinese context due to translation challenges. Generally, there are three expressions of bullying in the Chinese context—the traditional expression in spoken language is “欺负”, the recent official term is “欺凌” and another translation from Taiwan is “霸凌”. Therefore, the adoption of any of the three expressions could have advantages and tradeoffs in the survey, and thus, we decided not to use the expression “bullying” in our questions, but mainly described the behavioral phenomena. After a thorough review of relevant measurements, we adopted the newest SCS school bullying questions and designed a school bullying module with ten questions describing the behavioral actions related to bullying.

The being bullied, bullying others and witnessing bullying scenarios were inquired about in the survey separately. The question “In this year, has any student or your peer done the following things to you?” was used to inquire about the being bullied experience, while the question “In this year, have you done any of the following things to your classmates or peers?” was used to capture the bullying perpetration behaviors and the question “In this year, have you witnessed any of the following things happening?” was adopted to investigate the bullying witness experience. The same ten items were used with minor expression revisions to adjust to the three questions, six of them were related to traditional bullying behaviors and four of them were related to cyberbullying behaviors. The traditional bullying behaviors included “made fun of you, called you names or insulted you, in a hurtful way”, “spread rumors about you”, “threatened you with harm”, “pushed you, shoved you, tripped you, or spit on you”, “excluded you from activities on purpose” and “destroyed your property on purpose”. The four types of cyberbullying behaviors were “made fun of you, spread rumors about you on the internet, social media etc.”, “purposively posted your private information, photos or videos on the internet or social media”, “threatened or insulted you through social media, instant messaging (like wechat, QQ) or text messaging” and “purposively excluded you from online communication or in a game”. The answers to each of the items were “never”, “rarely”, “sometimes” or “frequently”. According to the repetitive nature of bullying [1], if a respondent chose “sometimes” or “frequently”, he/she was defined as being bullied (victim), bullying others (perpetrator) or witnessing bullying in that dimension, respectively. Otherwise, he/she was defined as “not being bullied”, “not bullying others” or “did not witness any bullying” in that dimension. And finally, if a respondent experienced or conducted any of the ten proposed intentional, negative actions repeatedly (sometimes or frequently), he or she was defined as being bullied or being a perpetrator.

#### 2.2.2. School Climate

The school climate included six variables—the type of school, whether it was a leading school in the region, self-evaluated relation with teachers, self-evaluated relation with classmates, perceived GPA ranking and boarding status. The school type had four options, primary school (grades 4–6), middle school, high school and vocational school which provides vocational and technical training for adolescents at high school ages, and the vocational school students are always perceived as “bad” students because of their lower academic performance. The leading school was a dummy variable indicating the quality of education of that school in that region. The self-evaluated relation with teachers and classmates was obtained by the question “Overall, what do you think of your relation with the teachers/classmates in your school?” The answers were measured by five Likert scales, ranging from one to five, indicating an increase of the relation quality from “very poor” to “very good”. The perceived GPA question was “Overall, how do you evaluate your study?” The answers were also measured by a five Likert scale, from “very poor (1)” to “very good (5)”. If a respondent lived on campus during weekdays, the boarding status was designated as one, otherwise, it was coded as zero.

#### 2.2.3. Control Variables

Some family attributes and demographic variables were included as controlled variables in the analysis. The family structure indicated the daily caregivers of the respondent and the answers were parents, father/mother or grandparents/other relatives. The father and mother’s education attainments were also included. Both of them ranked from one to five, representing “illiterate or primary school”, “middle school”, “high school or equivalent”, “college” and “graduate or above”. The perceived family’s overall socioeconomic status was obtained by the question “Think about your family’s social and economic ranking in your region, how would you like to rate it from one to five?” and the answers ranked from one to five, indicating a degree from low to high. The student’s ethnicity, gender and the geographical variation at the provincial level were also included.

### 2.3. Data Analysis

We first described the distribution of the sample. Then, the prevalence of bullying victimization, bullying perpetration and witnessing were reported. If a respondent had experienced any of the ten bullying behaviors as a victim, perpetrator or witness, he or she was defined as a bullying victim, bullying perpetrator or witness of bullying. Three logistic regression models were employed to analyze the correlation between school attributes, family features and the experience of being bullied, bullying others and witnessing bullying. Considering the fact that many bullying victims could also be perpetrators, we differentiated the respondents into four categories: the victim-only group, the perpetrator-only group, the ones who had been bullied and also bullied others (victim-perpetrator group), and the ones who had never experienced bullying as a victim or perpetrator (comparison group). A multinominal logistic regression model was used to explore the correlation between school climate variables and their potential roles in school bullying.

## 3. Results

### 3.1. The Characteristics of the Sample

Within the 3675 students in our sample, 16.08% of them were from Beijing, 12.46% were from Gansu, 12.82% came from Guangdong, 13.66% were from Guizhou, 21.44% were from Hunan, 10.56% were from Jiangsu and the last 12.98% were from Liaoning. Most of the students lived with their parents (73.28%), 9.9% of them mainly lived with one parent and 16.82% of them mainly lived with their grandparents or others. In terms of the parents’ education, the father’s education distribution ranked from middle school (31.35%), college (25.44%), high school or equivalent (24.24%), illiterate or primary school (10.07%), and graduate or above (8.9%). The mother’s education had a similar pattern, with a distribution of middle school (30.01%), college (25.01%), high school (22.29%), illiterate or primary school (14.34%), and graduate (8.35%). For the family’s perceived overall socioeconomic status within their region, 54.20% students believed their family ranked medium, 26.39% chose high to medium and 10.99% held the opinion of medium to low. The ones choosing high or low shared 4.57% and 3.84%, respectively. Moreover, 48.22% of our sample were boys, and 8.24% of them were minorities (Table 1).

In terms of school attributes, 37.77% of the respondents were primary school students, 27.76% of them were middle school students, 26.91% were high school students and the last 7.56% came from vocational school. About 34.45% of the respondents were from a leading school in their region in terms of education quality and about 18.18% of them were living on campus (boarding) during weekdays. 73.33% of the students indicated that they had “good” or “very good” relation with their teachers, 22.12% chose “medium” and only 4.55% of them said they had “poor” or “very poor” relation with their teachers. For relation with classmates, only 2.94% reported “poor” or “very poor” and 81.65% of them reported “good” or “very good”. 38.53% of the students perceived their study ranked “medium”, 31.37% reported “good”, 9.82% preferred “very good”, 14.75% indicated “poor” and 5.52% chose “very poor” (Table 1).

### 3.2. Prevalence of School Bullying

Overall, the incidences of self-reported bullying victimization, bullying perpetration and witnessing bullying were 26.1%, 9.03%, and 28.9%, respectively (Table 2). Within the 3675 students, 2654 of them had never experienced a bullying scenario in the last year. 271 of the 960 victims were also perpetrators, sharing 28.23% of all the victims. 271 of the 332 perpetrators were also bullying victims, sharing about 81.63% of the total perpetrators (Table 1).

Similar to the overall pattern, the prevalence of the ten bullying behaviors had the highest value for witnessing (except in relation to threatening), the medium value for being bullied and the lowest for bullying others. What’s more, the prevalence of traditional bullying was higher than the cyberbullying in general. Within the ten bullying behaviors, the ranking as victim was “make fun of” (18.8%), “spread rumor” (12.9%), “push, shove or kick” (8.73%), “destroy property” (6.39%), “social exclusion” (5.93%), “threaten” (5.85%), “social exclusion in cyber space” (5.01%), “make fun of in cyberspace” (4.27%), “threaten in cyberspace” (3.51%) and “spread rumor in cyber space” (3.18%). The ranking of the reported bullying of others was “make fun of” (5.61%), “spread rumor” (2.88%), “push or kick etc.” (2.80%), “threaten” (2.12%), “social exclusion” (2.07%), “destroy property” (1.88%), “social exclusion in cyberspace” (1.80%), “spread rumor in cyberspace” (1.50%), “threaten in cyberspace” (1.44%) and “make fun of in cyberspace” (1.44%). The ranking of witnessing bullying was “make fun of” (22.30%), “spread rumor” (15.10%), “push or shove or kick etc.” (14.70%), “destroy property” (11.10%), “social exclusion” (10.60%), “make fun of in cyberspace” (9.31%), “social exclusion in cyberspace” (8.68%), “spread rumor in cyberspace” (7.95%), “threaten in cyberspace” (7.86%) and “threaten” (1.74%) (Table 2).

### 3.3. The Correlation between School Climate and Bullying

The results from the three logistic regression models, with being bullied (victim), perpetration and witnessing bullying as dependent variables, were reported respectively in Table 3. All 3675 observations were used in the estimations and odds ratios were reported.

Overall, school bullying was more likely to occur among primary school students. In terms of being bullied, all of the middle school, high school and vocational school students had a lower likelihood of being bullied, and high school students had the lowest probability. The reported perpetration and witnessing had similar patterns, but the comparison between vocational school and primary school for perpetration, the comparison between middle school and primary school for witnessing, and the comparison between vocational school and primary school for witnessing were not significant. Contrary to the belief that students from good schools are better behaved, students in leading schools had a 38% higher likelihood of reporting being bullied and a 24% higher likelihood of reporting the witnessing of bullying. The reported perpetration in leading schools was also higher, though the effect was not statistically significant. The boarding status of the students was not statistically significant.

The relation with teachers, the relation with classmates and self-reported GPA ranking were protective factors, in general. With better relation with teachers, the student would have lower probability to report being bullied, to be a perpetrator and to witness bullying scenarios. The protective effects of classmates’ relation and self-reported GPA ranking were only significant for being a victim or being a witness.

For demographic variables, being a boy would mean a 60% higher probability of being a victim and a 90% higher probability of being a perpetrator. The ethnicity differences were not significant between the minority and the majority.

Almost all the family attributes played no significant roles in school bullying. The family’s perceived socioeconomic status and both parents’ education attainments had no significant effects on being a victim, being a perpetrator or being a witness. Compared with the students who mainly lived with their parents, the ones who mainly lived with a single parent or grandparents would have a higher probability of reporting the witnessing of bullying, but such differences were not significant for being bullied or bullying others. The geographical variances in school bullying were significant, especially for the self-reporting of being bullied.

The results of the multinominal logistic regression are reported in Table 4. The students were categorized into four groups, the bully-only group, victim-only group, bully-victim group and the non-bully-non-victim group (as the reference group). Compared with the students who were neither a victim nor a perpetrator, gender was the only significant predictor—being a boy had a higher probability of being a bully-only. The comparison between the non-bully-non-victim and victim-only, and the non-bully-non-victim and the bully-victim had similar patterns. The primary school students and students in leading schools had a higher probability of being a victim-only and bully-victim. The relation with teachers, relation with classmates and self-reported GPA ranking were protective factors. Being a boy was a risk factor and all the family attributes’ effects were not significant.

## 4. Discussion

In this paper, we examined the prevalence of school bullying in China using a national representative sample covering all types of schools related to adolescent education—the primary school, middle school, high school and vocational school. Information and reports on school bullying from the victims, perpetrators and witnesses were obtained and analyzed for cross-checking. The prevalence of victims’ reports of school bullying was 26.10%, while the prevalence of witness reports and perpetrator reports was 28.90% and 9.03%, respectively. It can be found that the prevalence of victim and witness reports was similar, which indicates that the results were relatively reliable. Previous surveys demonstrated that the victim reported incidence of being bullied was 8.6% among 11 to 18-year-old students [30] or 18.99% among middle school students in Guangdong [29], and 20% [27] or 25.7% among students in Beijing [7], much lower than the 44.6% in Xi’an [32] and much higher than another study’s 5.8% [8]. The reported incidences of being the bully perpetrator were much lower than the reported victimization, about half in most studies if they had this indicator, similar to our survey. One study of the reported incidence of witnessing bullying from Fujian Province was 27.91% [28], similar to our results. Therefore, we estimate that the prevalence of school bullying among Chinese adolescents is around 20%, and the reported perpetration should be lower than the reported victimization because the collectivism-oriented culture would not encourage such behavior. However, it should be noted that the prevalence of school bullying may vary in different regions of China, just as our analysis revealed, but did not deeply investigate. More detailed analyses are needed in future to truly understand the geographical variations. Also, the self-reported bullying of others was much lower than the reports of being bullied and witnessing bullying, indicating that bullying behavior is perceived as “wrong” by most of the youth, and this could provide implications for prevention, especially from the education perspective.

Our analysis reveals that school climate, especially the relation with teachers and relation with peers are significant protective factors in being bullied. The protective effects of relation with teachers on bullying others and witnessing bullying are also significant. Most of the prior studies support such conclusions cross-culturally in Iceland [46], The Netherlands [47], the United States [48] and Hong Kong [49], Macau [23]. But some cases show reversed effects, such as the situation in Colombia [50]. In a society with more diverse culture and ethnicity, such as the United States, the protective role of school climate may vary among students of different colors—it is only effective among white and Hispanic students, not among black students [51]. School climate contributes significantly to school bullying discourse [52], and considering the importance of contextual factors—especially the school context and climate [53]—whole school interventions with a strong emphasis on school climate are needed for the prevention of school violence and bullying [19].

Furthermore, boys are more likely to be involved in bullying behaviors as most prior studies demonstrated [24,32,54]. Unlike other studies using samples from a small region [32], or other countries [55], our analysis indicates that being involved in bullying behaviors does not vary according to most of the family characteristics, such as the family structure, parents’ education and the socioeconomic status.

This paper has at least three limitations. The first is that the nature of the cross-sectional design of this study cannot provide sound causality between school climate and school bullying. Secondly, our sample mainly covered the adolescents from urban areas using a convenient sampling method at the school level. Considering the huge urban-rural division in China, studies covering national representative students from rural areas are needed in the future. Thirdly, we did not include influencing factors like interpersonal relations, such as the relation with parents, and the community influences, which are proposed predictors of juvenile bullying behaviors in prior reviews [56], and a wider ecological perspective including more confounding factors is needed [12,13].

## 5. Conclusions

In this paper, the self-reported prevalence of school bullying behaviors from primary school, middle school, high school and vocational school students in seven provinces across China were reported. The incidence of self-reported victims of bullying was 26.10%, bullying others was 9.03% and witnessing bullying was 28.90%. The prevalence of bullying behaviors varied according to school attributes. Primary school students had higher bullying incidences and leading schools also had higher bullying incidences. School climate indicators—especially the relation with teachers, relation with classmates and perceived academic performance—were significant protective factors in being bullied in general. Boys were also more likely to be involved in bullying behaviors. Other family, socioeconomic and demographic factors’ effects were not significant.

## Figures and Tables

**Table 1:** The characteristics of the sample.

Variable	Freq.	Percent	Cumulative Percentage
Bully			
*Comparison*	2654	72.22	72.22
*Perpetrator*	61	1.660	73.88
*Victim*	689	18.75	92.63
*Both*	271	7.370	100
School Type			
*Primary*	1388	37.77	37.77
*Middle*	1020	27.76	65.52
*High*	989	26.91	92.44
*Vocational*	278	7.560	100
Leading School			
*NO*	2409	65.55	65.55
*YES*	1266	34.45	100
Relation with Teachers			
*Very Poor*	73	1.990	1.990
*Poor*	94	2.560	4.540
*Medium*	813	22.12	26.67
*Good*	1182	32.16	58.83
*Very Good*	1513	41.17	100
Relation with Classmates			
*Very Poor*	54	1.470	1.470
*Poor*	54	1.470	2.940
*Medium*	566	15.40	18.34
*Good*	1185	32.24	50.59
*Very Good*	1816	49.41	100
Perceived GPA			
*Very Poor*	203	5.520	5.520
*Poor*	542	14.75	20.27
*Medium*	1416	38.53	58.80
*Good*	1153	31.37	90.18
*Very Good*	361	9.820	100
Boarding			
*NO*	3007	81.82	81.82
*YES*	668	18.18	100
Minority			
*NO*	3372	91.76	91.76
*YES*	303	8.240	100
Boy			
*NO*	1903	51.78	51.78
*YES*	1772	48.22	100
Living with			
*Parents*	2693	73.28	73.28
*One Parent*	364	9.900	83.18
*Grandparents*	618	16.82	100
Father’s Education			
*Illiterate or Primary*	370	10.07	10.07
*Middle School*	1152	31.35	41.41
*High School or Equivalent*	891	24.24	65.66
*College*	935	25.44	91.10
*Graduate*	327	8.900	100
Mother’s Education			
*Illiterate or Primary*	527	14.34	14.34
*Middle School*	1103	30.01	44.35
*High School or Equivalent*	819	22.29	66.64
*College*	919	25.01	91.65
*Graduate*	307	8.350	100
Perceived Status			
*High*	168	4.570	4.570
*High to Medium*	970	26.39	30.97
*Medium*	1992	54.20	85.17
*Medium to Low*	404	10.99	96.16
*Low*	141	3.840	100
Province			
*Beijing*	591	16.08	16.08
*Gansu*	458	12.46	28.54
*Guangdong*	471	12.82	41.36
*Guizhou*	502	13.66	55.02
*Hunan*	788	21.44	76.46
*Jiangsu*	388	10.56	87.02
*Liaoning*	477	12.98	100
Total	3675	100	

**Table 2:** Prevalence of bullying victimization, perpetration and witnessing.

Variable	Victim	Perpetration	Witness
Bullying	26.10%	9.03%	28.90%
Make fun of	18.80%	5.61%	22.30%
Spread a rumor	12.90%	2.88%	15.10%
Push or kick etc.	8.73%	2.80%	14.70%
Threaten	5.85%	2.12%	1.74%
Exclude	5.93%	2.07%	10.60%
Destroy property	6.39%	1.88%	11.10%
Cyber make fun of	4.27%	1.44%	9.31%
Cyber rumor	3.18%	1.50%	7.95%
Cyber threat	3.51%	1.44%	7.86%
Cyber exclude	5.01%	1.80%	8.68%

**Table 3:** Logistic regressions ^+^: Being bullied, perpetration and witness (*N* = 3675).

Variable	Victim	Perpetration	Witness
School Type (primary as reference)			
*Middle*	0.44 *** (0.05)	0.55 *** (0.09)	0.91 (0.10)
*High*	0.31 *** (0.04)	0.34 *** (0.06)	0.64 *** (0.07)
*Vocational*	0.37 *** (0.08)	0.88 (0.28)	0.93 (0.18)
Leading School	1.38 ** (0.16)	1.31 (0.22)	1.24 * (0.13)
Relation with Teachers	0.75 *** (0.04)	0.75 *** (0.05)	0.90 * (0.04)
Relation with Classmates	0.75 *** (0.04)	0.87 (0.06)	0.74 *** (0.04)
Perceived GPA	0.86 *** (0.04)	0.89 (0.06)	0.91 * (0.04)
Board	1.25 (0.15)	1.16 (0.20)	1.05 (0.12)
Minority	0.83 (0.14)	1.01 (0.25)	0.96 (0.14)
Boy	1.60 *** (0.13)	1.90 *** (0.24)	1.09 (0.08)
Family (parents as reference)			
*Father/Mother*	1.06 (0.14)	1.10 (0.20)	1.40 ** (0.17)
*Grandparents*	1.08 (0.12)	1.22 (0.19)	1.41 *** (0.15)
Father’s Education	0.99 (0.06)	0.91 (0.07)	1.04 (0.05)
Mother’s Education	1.05 (0.06)	0.98 (0.08)	1.01 (0.05)
Status	1.02 (0.06)	1.05 (0.08)	1.02 (0.05)
Province (Beijing as reference)			
*Gansu*	2.24 *** (0.45)	1.20 (0.36)	2.99 *** (0.52)
*Guangdong*	3.96 *** (0.75)	1.30 (0.40)	3.92 *** (0.66)
*Guizhou*	2.55 *** (0.52)	1.48 (0.43)	2.75 *** (0.50)
*Hunan*	5.73 *** (1.01)	3.05 *** (0.76)	3.16 *** (0.51)
*Jiangsu*	2.89 *** (0.58)	0.77 (0.26)	2.02 *** (0.37)
*Liaoning*	2.25 *** (0.48)	1.32 (0.41)	1.95 *** (0.36)
Pseudo *R*^2^	0.148	0.117	0.067

+ Odds ratio reported; all three models’ overall tests were significant; standard errors in parentheses; * *p* < 0.05, ** *p* < 0.01, *** *p* < 0.001.

**Table 4:** Multinominal logistic regression of roles of school bullying (*N* = 3675).

Variable	Bully-Only	Victim-Only	Bully-Victim
School Type (primary as reference)			
*Middle*	−0.19 (0.35)	−0.78 *** (0.13)	−0.99 *** (0.20)
*High*	−0.55 (0.36)	−1.07 *** (0.13)	−1.65 *** (0.22)
*Vocational*	−0.48 (0.72)	−1.29 *** (0.27)	−0.42 (0.35)
Leading School	0.25 (0.33)	0.30 * (0.13)	0.38 * (0.19)
Relation with Teacher	−0.30 (0.16)	−0.26 *** (0.06)	−0.39 *** (0.08)
Relation with Classmate	−0.27 (0.16)	−0.32 *** (0.06)	−0.26 ** (0.08)
Perceived GPA	−0.02 (0.14)	−0.14 ** (0.05)	−0.19 * (0.07)
Board	−0.14 (0.41)	0.20 (0.14)	0.27 (0.19)
Minority	−0.34 (0.51)	−0.29 (0.20)	0.04 (0.28)
Boy	0.67 * (0.27)	0.39 *** (0.09)	0.80 *** (0.14)
Family (parents as reference)			
*Father/Mother*	0.35 (0.39)	0.08 (0.15)	0.05 (0.21)
*Grandparents*	0.26 (0.34)	0.03 (0.13)	0.19 (0.17)
Father’s Education	0.33 (0.18)	0.07 (0.06)	−0.17 (0.09)
Mother’s Education	−0.27 (0.18)	0.03 (0.06)	0.05 (0.09)
Status	0.16 (0.17)	0.02 (0.06)	0.04 (0.09)
Province (Beijing as reference)			
*Gansu*	0.16 (0.57)	0.97 *** (0.23)	0.32 (0.35)
*Guangdong*	−0.51 (0.71)	1.55 *** (0.22)	0.80 * (0.34)
*Guizhou*	0.97 (0.53)	1.18 *** (0.23)	0.39 (0.35)
*Hunan*	0.66 (0.50)	1.78 *** (0.20)	1.68 *** (0.29)
*Jiangsu*	0.28 (0.59)	1.39 *** (0.22)	−0.19 (0.41)
*Liaoning*	−0.20 (0.67)	0.85 *** (0.25)	0.56 (0.36)
Pseudo *R*^2^	0.127		

Standard errors in parentheses; the model was statistically significant; * *p* < 0.05, ** *p* < 0.01, *** *p* < 0.001.
